# Protective Elements of Mental Health Status during the COVID-19 Outbreak in the Portuguese Population

**DOI:** 10.3390/ijerph18041910

**Published:** 2021-02-16

**Authors:** Pedro Silva Moreira, Sónia Ferreira, Beatriz Couto, Mafalda Machado-Sousa, Marcos Fernández, Catarina Raposo-Lima, Nuno Sousa, Maria Picó-Pérez, Pedro Morgado

**Affiliations:** 1 Life and Health Sciences Research Institute (ICVS), School of Medicine, University of Minho, 4710-057 Braga, Portugal; pedromsmoreira@gmail.com (P.S.M.); soniamgaf@gmail.com (S.F.); beatriz.azevedo.couto@gmail.com (B.C.); mafaldagmsousa@gmail.com (M.M.-S.); marferrynx@gmail.com (M.F.); catarina.raposo.lima@gmail.com (C.R.-L.); njcsousa@med.uminho.pt (N.S.); mariapico231@gmail.com (M.P.-P.); 2 ICVS/3B’s, PT Government Associate Laboratory, 4710-057 Braga/Guimarães, Portugal; 3 Psychological Neuroscience Lab, CIPsi, School of Psychology, University of Minho, 4710-057 Braga, Portugal

**Keywords:** psychological, pandemic, isolation, Portugal, SARS-CoV-2, coronavirus

## Abstract

The outbreak of COVID-19 might produce dramatic psychological effects on individuals’ lives. In this study, we aimed to explore the elements that may reduce the negative effects on mental health of the quarantine period imposed by most governments during this worldwide crisis. We conducted an online survey to evaluate demographic, lifestyle and mental health variables in a sample of 1280 Portuguese individuals (79.8% females) with an average age of 37 years. We observed that factors related to living conditions, maintaining work either online or in the workplace, frequency of exercise and absence of previous psychological or physic disorders are protective features of psychological well-being (anxiety, depression, stress and obsessive-compulsive symptoms). Finally, the individuals previously receiving psychotherapeutic support exhibited better psychological indicators if they did not interrupt the process as a consequence of the outbreak. Our results indicate that the practice of physical exercise, reduced consumption of COVID-19 information and the implementation of remote mental healthcare measures might prevent larger impacts on mental health during the COVID-19 outbreak.

## 1. Introduction

The outbreak of the coronavirus disease 2019 (COVID-19) has originated a worldwide crisis with dramatic consequences for health, the economy and society [1]. Most governments are imposing quarantine periods, where the movements and contact between people are restricted to reduce the propagation of the disease. While the use of quarantine plays a critical role for the promotion of public health safety, it also has marked negative effects. In such an unprecedent scenario, people are restricted from maintaining contact with loved ones, their daily routines are dramatically changed and their working activities need profound adjustments [2]. These factors, together with the uncertainty, distress and fear related to the progression of the disease, are likely to increase the psychological burden, including anxiety [3], acute stress or depression [1,2]. Global stressors are known to produce marked detrimental effects on mental health. Natural disasters, for instance, are related to heightened risk for the development of negative psychological consequences [4]. Likewise, elevated psychological symptomatology has been repeatedly observed during the COVID-19 pandemic. A recent review of the scientific literature identified that a number of factors, including fear of illness and fear of negative economic consequences, are related to increased depression, anxiety and stress levels [5]. This issue has been observed in the general population and particularly in healthcare professionals and in individuals with pre-existing psychological disorders [6,7]. As in previous outbreaks, such as the H1N1 influenza, the repeated encouragement for cleaning and disinfection behaviors may conduct to an exaggeration of the likelihood and severity of contamination [8]. This scenario can lead to potential detrimental effects in obsessive-compulsive (OC) symptomatology, particularly in clinical populations [9], namely, due to the fact that (1) contamination and compulsive hand-washing rituals constitute one of the main manifestations of the symptoms of obsessive-compulsive disorder (OCD) and that (2) these patients tend to relapse in response to negative external or environmental cues [10], such as in a pandemic context. Notwithstanding, increases in OC symptoms following the first wave of the COVID-19 pandemic have also been described in non-clinical populations [11]. The abovementioned literature highlights the relevance of addressing these psychological variables in response to the pandemic and which factors may protect against psychological symptomatology.

While the absence of a quarantine can be even more detrimental to psychological health [12], the results from previous investigations highlight that the psychological impact of a quarantine is substantial and may subsist in the long term [1,2], being identified as a potential risk factor for the development of anger, stress and risky behavior, namely, online gambling [5]. Additionally, higher restrictions due to lockdown, together with a reduction in social contact, are associated with increased loneliness and psychological distress which are potential triggers for psychological symptomatology [13,14,15]. This impact is reported to be particularly pronounced among those with pre-existing mental health issues [16]. As such, human–human and also human–animal interactions are naturally positioned as potential predictors of individuals’ mental health during lockdown. For instance, animal ownership was reported to mitigate lockdown-related psychological symptomatology [17]. Recent publications have highlighted that an important aspect to consider when assessing the impact of quarantine on one’s mental health pertains to individuals’ housing conditions. Amerio and collaborators reported that living in small apartments, a low quality of indoor areas and the absence of green spaces are linked to increased incidence of depressive symptomatology [18]. In a context where the home becomes the workplace for many, physical housing conditions are thus likely to assume a greater impact on individuals’ everyday routines. While working from home does not seem to negatively impact job satisfaction or productivity—at least during the early phases of the lockdown—it does seem to correlate with the onset of physical and mental health issues [19]. Finally, lockdowns cause significant alterations in daily routines for most individuals, which may directly or indirectly produce an impact on individuals’ mental health. In line with this, the World Health Organization (WHO) has published a set of recommendations aimed to promote mental health during the pandemic. Among those, it is recommended for individuals to minimize exposure to news related to the pandemic which may be likely to increase anxiety and stress levels. On the other hand, individuals are encouraged to engage in healthy activities, such as exercising, which are likely to reduce the incidence of psychological symptomatology [20,21]. Considering that the determination of a quarantine is a necessary measure in most countries to face the COVID-19 pandemic, it is of utmost relevance to understand how we can minimize its negative psychological effects. In this investigation, we conducted a cross-sectional approach to understand whether specific variables related to living conditions during the quarantine period may have a protective role on the mental well-being of the Portuguese population. In line with the abovementioned review of the literature, the pandemic is expected to elicit increased expression of psychological symptomatology, namely, anxiety, depression, stress and obsessive-compulsive symptoms. Here, we intended to understand the impact of different elements including demographics, housing, daily activities and clinical history on psychological burden. In Portugal, the first COVID-19 cases were confirmed on 2 March 2020 and the Portuguese government imposed the emergency state (quarantine and social distancing measures) on 19 March 2020. At the time of the writing of the manuscript, Portugal had 23,392 confirmed and 231,737 suspected cases of COVID-19, with 1277 recoveries and 880 deaths (www.dgs.pt; accessed on 25 April 2020).

## 2. Materials and Methods

A sample of 1280 individuals (79.8% females) with a mean ± standard deviation age of 37.1 ± 12.1 years and education of 17.1 ± 3.4 years participated in this study (Table 1). An online survey was conducted to comprehensively characterize a set of demographic, social and mental health variables in a Portuguese sample during the outbreak of COVID-19. The survey started to be applied on 23 March 2020, four days after the declaration of the emergency state by the Portuguese government. Data were collected until the end of March. Participants were invited to collaborate through institutional e-mail, social media and local and national online newspapers. Only participants who were 18 years old or more were included in the study. Ethical approval was obtained from the Ethical Committee for Life Sciences of University of Minho (Braga, Portugal). Electronic informed consent was obtained from all the participants in which study goals were comprehensively explained. The study followed the Helsinki Declaration.

Demographic variables consisted of age (Age), gender (Gender) and years of education (Education). The survey included the Depression, Anxiety and Stress Scale with 21 items (DASS-21) [22] to evaluate depression, anxiety and stress symptoms experienced in the week before. Severe depression, anxiety and stress symptoms correspond to subscale scores higher than 10, 7 and 12, respectively. The Obsessive-Compulsive Inventory—Revised scale (OCI-R) [23] was also included to measure obsessive-compulsive (OC) symptoms in the previous month. An OCI-R score higher than 20 indicates the presence of OC disorder. The health status was also characterized regarding the existence of a previous diagnosis of physical (PhysicalDisorder) or psychological (PhychDisorder) conditions, whether the participants were receiving mental health support (MentalSupport) and, in that case, if the participants suspended their therapeutic process as a consequence of the pandemic (StopConsult). Questions pertaining to living conditions were also included: the existence of outdoor green spaces (Garden), number of individuals sharing the property (Housemates) and the presence of pets (Pets). Participants also indicated their current work status: whether they were working at the regular workplace (WorkRegular), teleworking (WorkOnline) or not working (NoWork). A set of questions was also included to characterize the frequency of daily activities, namely, the number of hours doing exercise (Exercise) and consuming information related to COVID-19 (MediaTime).

A sequence of multiple linear regression models was implemented to identify the significant predictors of mental health variables (DASS-21 and OCI). Dummy variables were created for the variables WorkStatus, Housemates, Garden and Pets. In addition, dichotomous variables were defined for the variables Exercise and MediaTime (0—less than one hour per day; 1—one or more hours per day). Finally, the models included variables related to health status to characterize the effect of a pre-existence of physical or psychological conditions (PhysicalDisorder and PsychDisorder, respectively). The models were adjusted for demographic variables (Gender, Age and Education). Statistical analysis was implemented with R version 3.6.1 (R Core Team, Auckland, New Zealand) [24]. The visualization of the regression models was produced with the sjPlot package [25]. The graphic representation of the models was produced using ggplot2 [26]

## 3. Results

The average scores for DASS-21 for depression, anxiety, stress and severity of obsessive-compulsive symptoms are presented in Table 2. Severe depression, anxiety and stress symptoms existed in 7.6%, 9.1% and 9.3% of the sample, respectively. Severe OC symptoms were present in 12.4% of the participants (Table 2).

**Figure 1:**
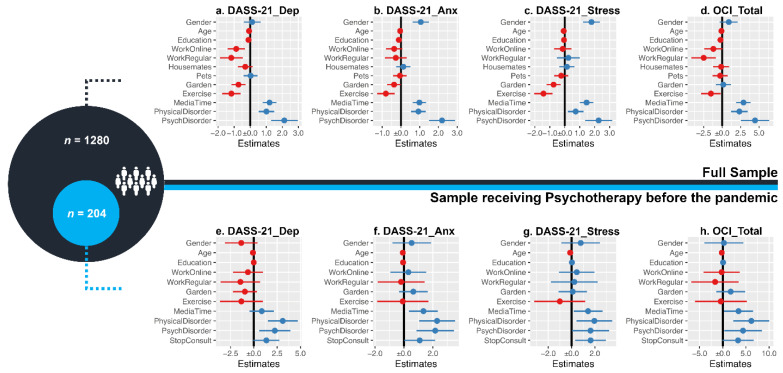
Representation of the multiple linear regression models. Each separate plot represents the estimates of a single linear regression model (the dependent variable Y is specified in the plot title). The vertical line corresponds to the boundary of statistical significance (i.e., no effect). Each row represents one predictor X: the dot is the regression coefficient, i.e., the estimated change in the outcome variable Y for each unit increase in the predictor X (blue and red correspond to positive and negative effects); the 95% confidence interval for the coefficient is represented as the segment line—if the interval does not cross the vertical line, X has a statistically significant effect on the dependent variable Y (considering a *p*-level of 0.05). The top of the figure (a-d plots) includes the regression models for the full sample (*n* = 1280); the predictors that were statistically significant in at least one model were included in the regression models for the subsample (f-h plots; *n* = 204; individuals receiving psychotherapeutic/psychiatric treatment before the pandemic—this sample was extracted from the initial sample of 1280 individuals). DASS-21: Depression, Anxiety and Stress Scale; Dep, Anx and Stress represent the different dimensions of DASS-21; OCI_Total: total score on the Obsessive-Compulsive Inventory—Revised version; Gender: dichotomous variable (0: male respondents); Exercise and MediaTime represent the daily time (1: one hour or more) practicing physical exercise and looking for COVID-19-related information, respectively; WorkOnline, WorkRegular, Garden, Pets and Housemates are dichotomous variables. PhysicalDisorder and PsychDisorder: presence of previous diagnosis of a physical or psychological condition, respectively; StopConsult: indicates whether the individual stopped receiving psychotherapeutic support as a consequence of the outbreak.

All the regression models were statistically significant (all *p*-values < 0.001). The proportion of explained variance ranged from 7% (R^2^_adj_ = 0.07; model g in Figure 1, Table 3 and Table 4) to 13% (R^2^_adj_ = 0.13; model f in Figure 1, Table 3 and Table 4). There was no evidence of multicollinearity between the variables (all Variance Inflation Factor values below 1.6).

The results from the linear regression models reveal a significant effect of Gender, with female participants reporting higher anxiety and stress (standardized B ≥ −0.126), lower Age (standardized B ≥ 0.124) and lower Education (standardized B ≤ −0.083, for depression, anxiety and OC symptoms), associated with higher psychological symptomatology. Housemates and Pets were not significant predictors of the outcome variables, while the existence of a garden was related to lower depression and stress (standardized B ≤ −0.055). Continuing to work (either remotely or in the workplace) was linked to lower depressive symptoms and marginally associated with significantly lower OC levels (standardized B ≤ −0.063). More Exercise (standardized B ≤ −0.062) and less MediaTime (standardized B ≥ 0.145) were associated with decreased symptom severity among the considered mental health variables. The presence of previously diagnosed psychological (standardized B ≥ 0.126) or physical disorders (standardized B ≥ 0.078) was associated with increased symptomatology for all variables. To further explore this finding, a complementary analysis in a subset of individuals receiving psychotherapy (*n* = 204) was conducted. The results from the linear regression models reveal that the suspension of the therapeutic process during the pandemic (StopConsult) was characterized by higher stress and anxiety (standardized B = 0.162 and standardized B = 0.140, respectively) (Figure 1 and Table 1).

## 4. Discussion

Our results provide elucidative insights about the predictors of mental well-being during the outbreak of COVID-19. Being male, active working, having a garden and practicing regular physical exercise seem to be relevant during this particular period. On the contrary, higher periods of media consumption seem to be related to poor mental health indicators.

Previous studies reported an adverse effect on mental health for younger Chinese participants (12 to 21 years) during the COVID-19 outbreak, mostly students [27]. They proposed that their routine was severely impacted by COVID-19 in comparison to older adults. Our results also point to a higher risk for younger individuals, despite the lack of children and adolescents in our sample. Other authors have also demonstrated that depression and anxiety levels were higher in individuals under 35 years of age [28]. Anxiety has been demonstrated to increase with increased concerns about the impact of the virus on economic and academic domains and decreased social support in college students [29], further supporting our findings. However, past research in China reported decreased distress in individuals under 18 years of age, but also enhanced distress for adults aged between 18 and 30 years, in line with our findings [30].

Our findings demonstrate that males are associated with less symptomatology. In accordance with such findings, post-traumatic stress disorder (PTSD), distress, anxiety and depression symptoms were higher in Chinese women during the pandemic [30,31,32]. Contrary to our results, higher education in Chinese individuals was associated with increased distress during the effects of COVID-19 [30]. These authors suggested that more educated people are more aware of their health. However, the previous literature supports our results [2,33].

Our results agree with past research demonstrating that working at home or working without restrictions when compared to not working was associated with better mental health, life satisfaction and lower distress in Chinese individuals during the COVID-19 pandemic [34]. On the other hand, healthcare workers presented elevated values of insomnia/poor sleep quality, fear, anxiety, depression and OC symptoms due to COVID-19 [28,35,36]. Thus, further analyses should explore the effect of riskier job categories on mental health.

In line with our findings, recent reports demonstrated an association between frequent social media exposure or access to COVID-19 information and increased levels of depression and anxiety in the Chinese population during the COVID-19 pandemic [27,28,37]. Worry feelings were also associated with exposure to COVID-19 information on social media and television and in journals in India [38]. The adoption of lifestyle changes such as increased resting, relaxing and exercising time was associated with lower stress scores [39]. However, groups with highly physically active individuals reported reduced life satisfaction. These individuals might have been more affected by the confinement impositions in China [34]. In Portugal, the population was allowed to go outside for short periods during the first emergency state for exercise and child outdoor activities. Thus, physical activity is only limited by social distancing measures. Moreover, also in agreement with our results, gardening is related to a positive impact on mental health because it involves more physical activity and better access to healthy food [40].

We did not find statistically significant effects regarding the number of housemates. Previous authors point to a negative effect of living with families in depression scores [36] and increased PTS while living with more housemates [27] during the pandemic in China. However, other authors reported a beneficial effect for anxiety of living with parents in Chinese college students during the outbreak [29]. Herein, we did not discriminate the housemates belonging to the family. Thus, future analyses should investigate in more detail the housemates’ characteristics (e.g., age range, relationships and infection vulnerability). Living with people more susceptible to be infected [12,32] or taking care of younger or elderly family members while working remotely might have an impact on mental health.

Moreover, Chinese individuals with non-psychiatric diseases had higher risk for insomnia, depression and OC symptoms during the pandemic [36], and chronic diseases were associated with reduced life satisfaction [34] and enhanced levels of PTS, anxiety, depression and stress [27]. These results agree with our findings pointing to the diagnostic of a psychical/mental disorder as a risk factor for mental health.

One point of particular interest for mental health providers relates to the negative effects of the discontinuation of therapeutic processes on measures of acute psychological symptomatology [41,42]. Past research focusing on Chinese healthcare workers reported more mental disturbances for individuals with reduced access to printed/online psychological help resources [43]. It raised the need for the adoption of strategies aimed to deliver psychological support through the use of remote tools, including videoconferences or other web-based technologies [1,44]. Through the use of such approaches, the negative effects of a quarantine on psychological well-being can be ameliorated.

Previous longitudinal investigations of mental health parameters during the pandemic in China did not report changes in anxiety, depression and stress scores (DASS-21) but demonstrated a statistically significant but clinically meaningless decrease in PTS values. Indeed, the PTS score in the two timepoints was above the cut-off value. Augmented washing and avoidance behaviors in the face of putative contamination were associated with decreased PTS and DASS-21 scores [38]. The authors suggested that the implementation of lockdown and public health measures might explain these outcomes because they might have increased the population’s confidence and safety feelings [30]. The prevalence of depression, anxiety and impulsivity disorders in Portugal in 2014 was 7.9%, 16.5% and 3.5%, respectively (www.dgs.pt, accessed on 25 April 2020). OC disorder has a prevalence of 2–3% [45]. Our results of severe OC symptoms in 12.4% of the sample might indicate an increase in OC symptoms during the pandemic, but this needs to be confirmed by longitudinal analyses. It is important to study the evolution of OC symptoms over time to prevent higher incidence of OC disorder during and after the pandemic [1]. Indeed, some studies suggest negative psychological effects months or years after [2].

Our results are limited by the cross-sectional design. This aspect precludes us from drawing clear cause-and-effect relationships between the variables. As such, we cannot rule out the possibility that the reported associations between the target variables with specific elements of participants’ daily living were already present before the pandemic, even if they have been amplified by the outbreak of COVID-19. Another point to mention pertains to the age distribution of our sample, which is younger and has a greater proportion of women than the average Portuguese population (www.ine.pt; accessed on 25 April 2020) and does not include children and adolescents. A final note that is worth mentioning pertains to the modest percentage of explained variance of the target variables (0.07 ≤ R^2^adj ≤ 0.13). This indicates that other aspects not considered in our analysis are relevant for the observed variation of psychological symptomatology. Again, given the cross-sectional design of this investigation, we cannot rule out other aspects such as variations in the propensity of psychological symptomatology and other contextual events not related to the pandemic, among others. As such, future studies may benefit from the inclusion of less varying psychological variables (such as trait anxiety) in regression models such as the ones we present in this report. Furthermore, the use of longitudinal approaches is desired to further clarify the nature of such relationships.

Nonetheless, our findings agree with a recent review of previous quarantine periods unrelated to COVID-19 [3] and replicate the major results in the Chinese population for preventive factors of mental health during the COVID-19 outbreak: (1) demographic (male gender, older age and higher education); (2) lifestyle (active working, more exercise, use of green spaces and less exposure to COVID-19 information); (3) clinical (absence of physical/mental health disorders and continuity of mental healthcare). These outcomes align with recent recommendations to protect mental health during the pandemic and prevent long-term effects [1,41].

## 5. Conclusions

In this cross-sectional study, we observed that living conditions, lifestyle, maintaining work (either online or in the workplace) and the presence of a previous psychological or physic disorder are associated with psychological well-being during the pandemic. Furthermore, the need for discontinuing a psychotherapeutic process as a consequence of the pandemic was significantly related to the scores on such psychological variables.

## Figures and Tables

**Table 1:** Description of the survey demographic, social and mental health variables, and the statistical results for the linear regression models.

	Percentage	*b* DASS-21 Depression	*b* DASS-21 Anxiety	*b* DASS-21 Stress	*b* OCI
Gender (%female|%male)	79.84|20.16	0.121 (0.268)	1.054 (0.222)	1.786 (0.291)	0.9 (0.623)
Age (years)	37.10 ± 12.05	−0.073 (0.01)	−0.044 (0.008)	−0.072 (0.01)	−0.094 (0.022)
Education (years)	17.13 ± 3.40	−0.105 (0.033)	−0.113 (0.027)	−0.053 (0.035)	−0.222 (0.076)
Work status					
%WorkOnline	62.89	−0.87 (0.274)	−0.362 (0.227)	−0.141 (0.297)	−1.184 (0.636)
%WorkRegular	14.77	−1.178 (0.364)	−0.274 (0.301)	0.236 (0.395)	−2.489 (0.845)
%NoWork	22.34				
Number of housemates (% ≤ 1|% > 1)	32.9|67.1	−0.307 (0.239)	0.128 (0.198)	0.145 (0.259)	−0.139 (0.555)
Pets (%yes|%no)	52.11|47.89	0.029 (0.223)	−0.056 (0.184)	−0.238 (0.241)	−0.271 (0.517)
Garden (%yes|%no)	47.73|52.27	−0.731 (0.225)	−0.37 (0.186)	−0.748 (0.244)	0.161 (0.522)
Exercise (% < 1 h|% ≥ 1 h)	82.97|17.03	−1.171 (0.29)	−0.806 (0.24)	−1.425 (0.314)	−1.51 (0.672)
Media time (% < 1 h|% ≥ 1 h)	58.28|41.72	1.205 (0.217)	0.985 (0.18)	1.459 (0.236)	2.886 (0.504)
Physical disorder (%yes|%no)	66.95|33.05	1.011 (0.246)	0.931 (0.203)	0.727 (0.266)	2.337 (0.57)
Psychological disorder (%yes|%no)	92.81|7.19	2.125 (0.425)	2.19 (0.351)	2.266 (0.46)	4.457 (0.986)
Stop psychological consultations (%yes|%no) ^a^	89.61|10.39	1.343 (0.708)	1.09 (0.543)	1.631 (0.672)	3.297 (1.709)

Data correspond to mean ± standard deviation. ^a^ Subsample receiving psychological support (*n* = 204). ^b^ DASS-21, Depression, Anxiety and Stress Scale; OCI, Obsessive-Compulsive Inventory. Values in parentheses represent the standard error for the unstandardized regression coefficients.

**Table 2:** Description of depression, anxiety, stress and obsessive-compulsive symptoms.

	Mean ± Standard Deviation	Range	%Normal	%Mild	%Moderate	%Severe	%Extremely Severe
DASS-21 depression	3.73 ± 4.03	0–21	68.91	12.34	11.17	3.52	4.06
DASS-21 anxiety	2.64 ± 3.34	0–20	71.56	12.50	6.80	3.98	5.16
DASS-21 stress	6.06 ± 4.40	0–21	69.30	11.02	10.39	6.80	2.50
	Mean ± standard deviation	Range	%Not severe			%Severe	
OCI total	10.26 ± 9.14	0–65	87.58			12.42	
OCI washing	2.74 ± 2.64	0–12					
OCI checking	1.24 ± 1.75	0–11					
OCI ordering	2.25 ± 2.46	0–12					
OCI hoarding	1.63 ± 2.14	0–12					
OCI obsessing	1.70 ± 2.44	0–12					
OCI neutralizing	0.69 ± 1.47	0–12					

DASS-21, Depression, Anxiety and Stress Scale; OCI, Obsessive-Compulsive Inventory.

**Table 3:** Description of the regression models for the full sample.

	Model a: EADS_Dep			Model b: EADS_Anx			Model c: EADS_Stress			Model d: OCI_Total		
Predictors	B	SE	Beta	B	SE	Beta	B	SE	Beta	B	SE	Beta
(Intercept)	8.60 ***	0.75	0	5.02 ***	0.62	0	7.90 ***	0.82	0	16.07 ***	1.75	0
Gender	0.12	0.27	0.01	1.05 ***	0.22	0.13	1.79 ***	0.29	0.16	0.9	0.62	0.04
Age	−0.07 ***	0.01	−0.22	−0.04 ***	0.01	−0.16	−0.07 ***	0.01	−0.2	−0.09 ***	0.02	−0.12
Education	−0.11 **	0.03	−0.09	−0.11 ***	0.03	−0.11	−0.05	0.04	−0.04	−0.22 **	0.08	−0.08
WorkOnline	−0.87 **	0.27	−0.1	−0.36	0.23	−0.05	−0.14	0.3	−0.02	−1.18	0.64	−0.06
WorkRegular	−1.18 **	0.36	−0.1	−0.27	0.3	−0.03	0.24	0.39	0.02	−2.49 **	0.84	−0.1
Housemates	−0.31	0.24	−0.04	0.13	0.2	0.02	0.14	0.26	0.02	−0.14	0.56	−0.01
Pets	0.03	0.22	0	−0.06	0.18	−0.01	−0.24	0.24	−0.03	−0.27	0.52	−0.01
Garden	−0.73 **	0.22	−0.09	−0.37 *	0.19	−0.06	−0.75 **	0.24	−0.09	0.16	0.52	0.01
Exercise	−1.17 ***	0.29	−0.11	−0.81 ***	0.24	−0.09	−1.43 ***	0.31	−0.12	−1.51 *	0.67	−0.06
MediaTime	1.20 ***	0.22	0.15	0.98 ***	0.18	0.15	1.46 ***	0.24	0.16	2.89 ***	0.5	0.16
PhysicalDisorder	1.01 ***	0.25	0.12	0.93 ***	0.2	0.13	0.73 **	0.27	0.08	2.34 ***	0.57	0.12
PsychDisorder	2.13 ***	0.42	0.14	2.19 ***	0.35	0.17	2.27 ***	0.46	0.13	4.46 ***	0.99	0.13
Overall	F_(12, 1266)_ = 14.1, *p* < 0.001,			F_(12, 1266)_ = 14.5, *p* < 0.001,			F_(12, 1266)_ = 15.6, *p* < 0.001,			F_(12, 1266)_ = 8.8, *p* < 0.001,		
	R^2^ = 0.12, R^2^_adj_ = 0.11			R^2^ = 0.12, R2_adj_ = 0.11			R^2^ = 0.13, R^2^_adj_ = 0.12			R^2^ = 0.08, R^2^_adj_ = 0.07		

* *p* < 0.05, ** *p* < 0.01, *** *p* < 0.001. B: unstandardized coefficients, SE: standard errors, Beta: standardized coefficients, R^2^adj: adjusted R-Square

**Table 4:** Description of the regression models for the sample receiving psychotherapy.

	Model e: EADS_Dep			Model f: EADS_Anx			Model g: EADS_Stress			Model h: OCI_Total		
Predictors	B	SE	Beta	B	SE	Beta	B	SE	Beta	B	SE	Beta
(Intercept)	8.44 ***	2.13	0	3.84 *	1.63	0	7.74 ***	2.02	0	11.57 *	5.14	0
Gender	−1.36	0.88	−0.1	0.53	0.68	0.05	0.78	0.84	0.06	0.24	2.13	0.01
Age	−0.10 **	0.03	−0.24	−0.08 ***	0.02	−0.25	−0.12 ***	0.03	−0.3	−0.21 **	0.07	−0.22
Education	−0.01	0.1	−0.01	−0.06	0.07	−0.05	0.03	0.09	0.02	0.07	0.23	0.02
WorkOnline	−0.64	0.82	−0.07	0.31	0.63	0.04	0.44	0.78	0.05	−0.24	1.98	−0.01
WorkRegular	−1.45	1.07	−0.11	−0.2	0.82	−0.02	0.24	1.02	0.02	−1.65	2.59	−0.05
Garden	−0.97	0.66	−0.1	0.65	0.5	0.09	0.1	0.62	0.01	1.71	1.59	0.07
Exercise	−1.35	1.17	−0.08	−0.09	0.9	−0.01	−1.01	1.11	−0.06	−0.43	2.83	−0.01
MediaTime	0.84	0.66	0.09	1.35 **	0.51	0.18	1.42 *	0.63	0.16	3.39 *	1.59	0.15
PhysicalDisorder	3.10 ***	0.82	0.32	2.29 ***	0.63	0.3	1.96 *	0.77	0.21	6.17 **	1.97	0.27
PsychDisorder	2.25 **	0.85	0.21	2.16 **	0.65	0.26	1.62 *	0.81	0.16	4.38 *	2.06	0.18
StopConsult	1.34	0.71	0.13	1.09 *	0.54	0.14	1.63 *	0.67	0.17	3.3	1.71	0.14
Overall	F_(11, 192)_ = 3.0, *p* < 0.001,			F_(11, 192)_ = 3.7, *p* < 0.001,			F_(11, 192)_ = 3.3, *p* < 0.001,			F_(11, 192)_ = 2.4, *p* = 0.009,		
	R^2^ = 0.15, R^2^_adj_ = 0.10			R^2^ = 0.18, R^2^_adj_ = 0.13			R^2^ = 0.16, R^2^_adj_ = 0.11			R^2^ = 0.12, R^2^_adj_ = 0.07		

* *p* < 0.05, ** *p* < 0.01, *** *p* < 0.001. B: unstandardized coefficients, SE: standard errors, Beta: standardized coefficients, R^2^adj: adjusted R-Square

## Data Availability

The data is available at https://osf.io/3rztv/.
